# Investigation on Motorcyclist Riding Behaviour at Curve Entry Using Instrumented Motorcycle

**DOI:** 10.1155/2014/968946

**Published:** 2014-01-12

**Authors:** Choon Wah Yuen, Mohamed Rehan Karim, Ahmad Saifizul

**Affiliations:** Centre for Transportation Research, Faculty of Engineering, University of Malaya, 50603 Kuala Lumpur, Malaysia

## Abstract

This paper details the study on the changes in riding behaviour, such as changes in speed as well as the brake force and throttle force applied, when motorcyclists ride over a curve section road using an instrumented motorcycle. In this study, an instrumented motorcycle equipped with various types of sensors, on-board cameras, and data loggers, was developed in order to collect the riding data on the study site. Results from the statistical analysis showed that riding characteristics, such as changes in speed, brake force, and throttle force applied, are influenced by the distance from the curve entry, riding experience, and travel mileage of the riders. A structural equation modeling was used to study the impact of these variables on the change of riding behaviour in curve entry section. Four regression equations are formed to study the relationship between four dependent variables, which are speed, throttle force, front brake force, and rear brake force applied with the independent variables.

## 1. Introduction

It is well recognized that road safety is a public health problem as road traffic accidents are among the eight leading causes of death worldwide, according to Loo et al. [1]. Malaysia, as well as other countries that have high volumes of motorcycles, is facing high motorcyclist fatalities. Motorcyclists represent an important concern from a road safety perspective [2]. Considerable efforts have been taken to reduce the number of these fatalities on the road every year; yet the number of fatal accidents that involve motorcyclist still stands high [3–5].

Motorcycle crashes continue to be a problem in both developing and developed countries. According to Radin Umar et al. [6], a preliminary investigation on motorcycle fatalities showed that riding a motorcycle is 17 times more dangerous than driving a passenger car. The huge number of motorcycles on road may cause the high rates of motorcycle accidents. In Malaysia, motorcycle crash is a serious issue as the fatality number and vehicle accident that involve motorcycle are standing high. When observed in Table 1, one should find that most fatal number in traffic accident is motorcycle. This mode of transport constitutes more than half, which is over 58% of total fatality in years 2006 to 2010. Besides, motorcycles also become the most dominant mode involved in vehicle accident, which recorded an average of over 40% from the total vehicles involved in years 2006 to 2010 (as shown in Table 2). These two figures reflected the fact that motorcyclists are more involved in traffic crash and get injured or killed in vehicle crash compared to other modes of transport. It can be concluded that in the majority of traffic accidents in which they are involved, motorcyclists tend to be the victims of errors made by other road users. Thus, safety of this form of transportation is an important issue.

Mixed traffic that contains motorized and nonmotorized vehicles becomes more common in urban area. Mixed traffic flow contains standard vehicle types such as passenger cars, buses, and trucks and also nonstandard vehicles such as motorcycles and bicycles. Cho and Wu [7] have done a research on studying the motorcycle traffic flow. In their work, they pointed out that motorcycle traffic flow is greatly influenced by driver characteristics, vehicle interactions, and the external environment. In other words, motorcyclist riding behavior varies and is influenced by an external environment factor. Thus, it is important to examine the impact of external environment towards motorcycle traffic flow especially on the motorcyclist riding behavior.

## 2. Malaysia Motorcycle Crash Study

**Figure 1:**
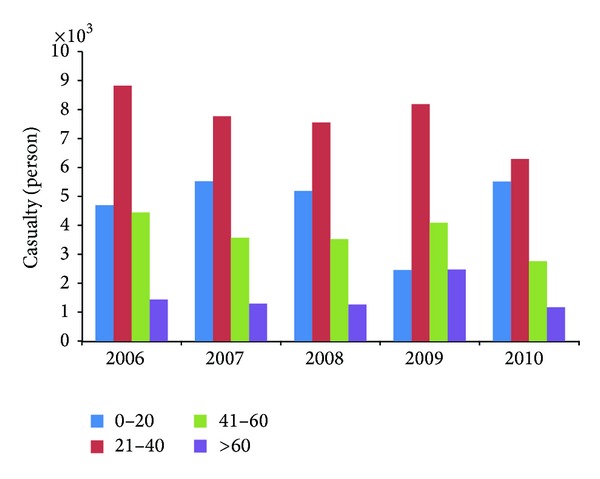
Motorcyclist casualty by age group in Malaysia.

In a more detailed study on Malaysia motorcycle accident data (as shown in Figure 1), it was found that the number of casualties for riders of age group 21–40 recorded the highest number of casualties followed by riders of age group 0–20, while elder group riders are recorded as the least casualties compared to other age groups. It seems like riders with lower age group tend to involve and get injured in motorcycle accident compared to riders from the elder group. A similar study on the accident characteristics of injured motorcyclists in Malaysia was done by Pang et al. [8]. They found that most of the injured motorcyclists were young, novice riders of less than 3 years licensure, and male. However, the study done by Chang and Yeh [9] showed a different result. Chang's investigation showed that female riders are more likely to be involved in an accident compared to male riders. Furthermore, the study also found that young male riders' involvement in more accidents may be due to risky behaviours, whereas young female riders' might be due to other latent factors such as lack of experience and skills. Speeding violations were found to be the most common abhorrent behavior among the motorcyclists [10]. Young motorcycle riders may not be aware of the errors and violations they make and also the consequences of their negligent behaviour. This may explain why most of the accidents involved young riders. In short, all the study, data, and facts above reflected one truth, which is that motorcycle is proved to be a transport mode that tends to be involved in road accident and most exposed to the danger of losing their valuable life. Actions should be taken to improve the safety of this mode of transport.

Motorcycle accident has been an active research topic in recent years. Motorcyclists represent an important concern from a road safety perspective [2]. There is a need to identify other related factors that cause motorcycle accidents before action can be taken to reduce the number of accidents. Research on the crash risk of motorcyclists has investigated a variety of issues, such as rider attributes, motorcycle characteristics, roadway, environmental and traffic factors, and overexposure of motorcycles at intersections [11]. Various studies showed that age [12–16], riding manner such as speeding [10, 17–19], traffic road environment [20–23], gender [9], and ride experience [8] were among the reasons involving motorcycles in accidents. According to Yeh and Chang [24], motorcycle riders have an increased likelihood of accidents compared with other motor vehicle drivers. Broughton et al. [17] found that riders who ride at unsafe high speed will have a higher crash probability. Furthermore, a study also showed that greater proportions of both young and elderly drivers have led to higher death rates [14]. Accident statistics also proved that young drivers have significantly higher accident violation rates than older drivers. This is further supported in Wong et al.'s [15] study, where they had identified young motorcyclists as a high-risk population in causing motorcycle accidents. Road environment is a characteristic that a traffic engineer can intervene to improve and prevent crashes from occurring [22]. Certain road conditions in local roads and off-road sites are found to be hazardous for motorcycle riders. In particular, potholes, gravel, leaves/branches, and deterioration of the road surface can easily lead to motorcyclists losing control of their motorcycles [25].

As one should know, riders would ride in extra careful manner when they ride in some hazardous locations such as slippery road, curve section, or down slope section. A study case on level terrain curve riding was performed using the instrumented motorcycle. The design of this study is to investigate the change in riding behaviour when riders ride at a straight road path leading into a curve section. This paper studies how riders would react and respond, as well as how they would adjust their riding behaviour, when they ride approaching a curve section. This paper should provide a general study and knowledge for riding behaviour study on curve section. By conducting such riders' behaviour studies, traffic engineers, law enforcers, and policy makers may take effective actions to reduce the number of accidents and therefore save valuable human life.

## 3. Methodology

### 3.1. Instrumented Motorcycle

Subjects performed all experimental sessions within an instrumented Honda Wave 110. This motorcycle contained various sensors apparatus such as steering angle sensor, throttle sensor, pedal position sensor which is linked to the brake pedal, steering folk, and throttle roll. The registered speed and distance travelled were recorded using GPS loggers that were fitted inside the motorcycle storage box. A GPS antenna is installed at the highest point of the motorcycle to ensure that the GPS signal can be received clearly throughout the experimental riding process. The following data were collected from the GPS antenna. All data were sampled at 2 Hz, digitised, and stored on a compact flash card which was fitted into the video logger.

On the instrumented motorcycle, a sensor was installed both at the front brake and rear brake pedal to measure the intensity of brake force applied. Once the brake pedal is applied, the braking force measured is converted and presented in percentage reading. Both brake system values are recorded and combined as one overall brake value. In this case, the higher value of the rear brake and front brake will be taken as the overall brake value. In other words, the brake value is taken as the maximum value from either the rear brake or front brake intensity force. Throttle force value will also be obtained as a percentage value, where the voltage generated from the sensor will be converted to percentage. This parameter is important as we need to know under what situation and circumstances will the rider apply the throttle and at what degree of throttle intensity to accelerate and increase their riding speed.

**Figure 2:**
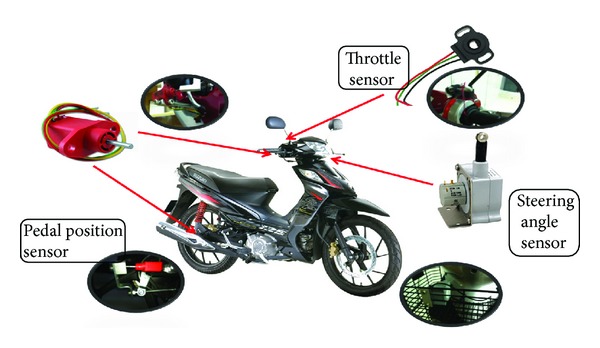
Instrumented motorcycle with various sensors location.

Besides, there were three bullet cameras used to capture real-time traffic image while running the test. One bullet camera is mounted on the riders' helmet to provide their real-time view image. Another bullet camera is mounted in front of the motorcycle to capture the front traffic stream. The third bullet camera is mounted on the back of the motorcycle to capture the real-time back traffic stream. All video streams will be transferred and stored in a video logger. The arrangements of sensors are as shown in Figure 2.

### 3.2. Subjects Selection

Riders with motorcycle riding experience were invited to participate in this study. All recruited riders were voluntary participants in the project. The criteria for rider selection are as follows.

- They need to have a valid motorcycle B2 class license.
- They ride a motorcycle regularly, to be precise, at least once a week.
- They should ride at the motorway on a regular basis.

The mean age of participants is 31.28, mean riding experience is 13.18, and mean kilometers' riding per week is 164.21. Riders were requested to ride with their usual riding manner and behaviour on the instrumented motorcycle. Subjective measures and riders' personal details were taken from a simple questionnaire. Basic information and personal details of riders were taken for record and analysis purposes. The subjective measures and personal information which were included in the survey form were rider's name, gender, occupation, age, license class owned, years of riding experience, and average weekly travel mileage.

### 3.3. Study Site Selection

**Figure 3:**
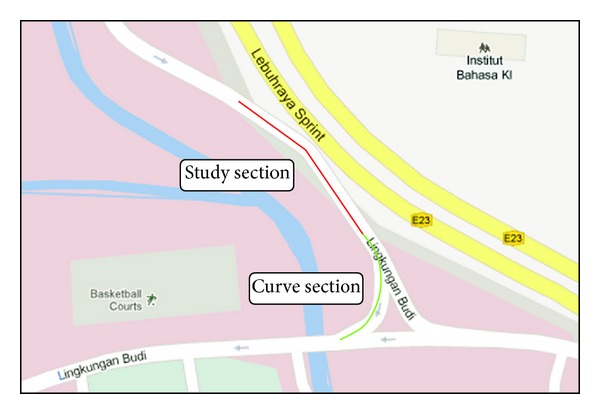
Plan view of study site.

The selected level terrain curve section is a one-way traffic roadway with a transition curve of varying radii, located inside the main campus of University of Malaya, Malaysia. The plan view of the study area is shown in Figure 3. Transition curves provide a gradual change of degree and easier riding in going from the tangent to a full curvature. The curve section is defined by the radii of curve (RC) value, which is measured in meter. The closer a curve radius value to 0 m indicates more curve of that particular section, contrary the straight road gives maximum corner radius value which is 2000 m. In this study, a section is defined as a curve when the section's RC is below 200 m. The chosen site has good pavement surface with free flowing traffic. Besides, there should not be any other obstructions or interferences such as a hump or junction at a distance of 100 m before the studied curve section. This criterion is very important as we wanted to make sure that riders can ride at a free speed at the initial distance (100 meters) from the curve entry, which also enables us to obtain a more accurate riding behaviour result, completely free from other outside interference.

A total number of 67 sets of data were collected in the study. Markers were made at the important points such as the initial point (100 m before curve entry) and on curve entry point on the virtual route map for analysis purposes. The traffic data were collected starting at the 100 m point before the curve entry till the curve entry point. The reason for taking 100 m before the curve entry as a starting point is because this is an appropriate distance to cover for riders travelling at a free speed and a good distance to observe when a curve section starts to take an impact on reducing riders' speed and influencing riding behaviour in response to the entering of the curve section.

### 3.4. Experimental Procedures

Each experimental riding session was conducted according to the following protocol: on arrival at the starting point, the subjects were briefed on the route map. Subjects were instructed to ride on a motorway route within the campus of University of Malaya, Malaysia. The subjects were asked to ride with their usual and natural riding behaviour. All the riding sessions were performed in the morning period, when the weather was clear and the roadway was in dry condition. Upon completion of the journey, the subjects were asked to complete a simple questionnaire. Subjective measures, such as age, riding experience, and others, were taken from the simple questionnaire for record and analysis purposes. The traffic data collected in the experimental run was recorded and stored in a compact flash memory card. The data was then further analysed using system manufacturer analysis software. From the analysis software, data such as travel speed, throttles applied, brakes applied, and GPS positions of the motorcycle were extracted.

## 4. Results and Discussion

### 4.1. Speed and Braking Behaviour Study

**Figure 4:**
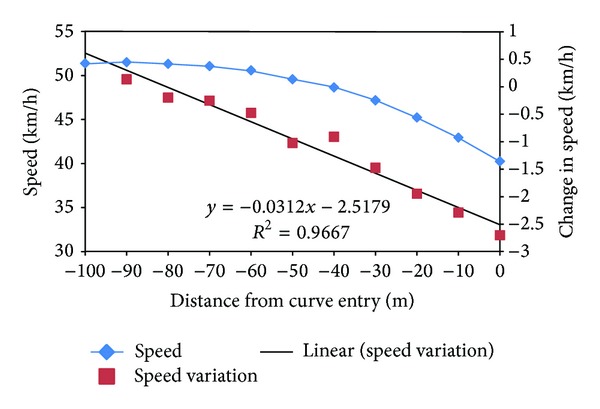
Speed profile and change rate before curve entry.

Figure 4 shows the speed profile throughout the whole riding section before curve entry. From the figure, travel speed was found reduced as they ride approaching the curve section. However, the speed change rate varied with the change in distance from the curve entry. At the initial distance (100 m before curve entry), the riding speed was recorded as 51.406 km/h and had reduced to 40.272 km/h right at the curve entry point. Interestingly, as shown in Figure 4, the speed was found reduced at a constant rate and can be predicted by (1) as shown below:

(1) $$\begin{array}{c} \text{Change}\;\text{in}\;\text{Speed}=-0.0312\;\text{Distance}-2.5179. \end{array}$$

**Figure 5:**
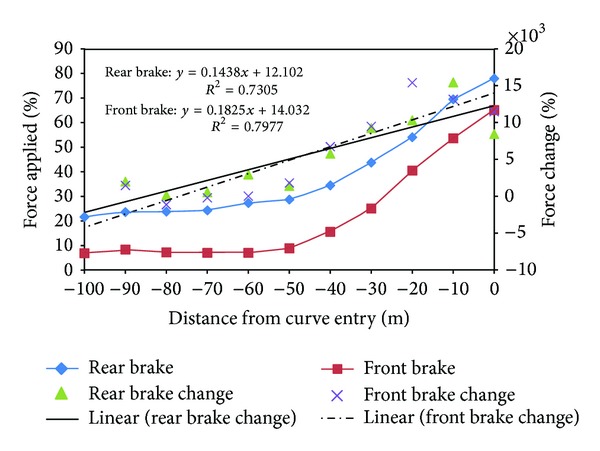
Rear brake and front brake applied profile.

It was found that speed decreases in higher rate, which is more than −1 km/h speed drop starting from the point −40 m from the curve entry. This was due to a higher brake intensity force applied at the same distance point (refer to Figure 5), where riders tended to adjust their riding to a comfortable speed before entering the curve section. In short, the results showed that the closer the riding towards the corner section is, the lower the speed recorded is and the higher rate the speed reduced is.

In the brake behaviour study, there are two braking data collected through the instrumented motorcycle, which are rear brake intensity value and front brake intensity value. Generally, as shown in Figure 5, it was found that riders tend to apply more rear brake compared to front brake at the whole riding procedure. A nonparametric analysis test, Wilcoxon test, was performed to study whether there was a significant difference between rear brake and front brake applied. From the test results, it was found that *Z* = −11.566, *N* = 641. A two-tailed analysis was carried out and found *P* < 0.001, which is significant at *P* < 0.05. In short, one can conclude that the rear brake and front brake force applied are significantly different. Besides, both braking lines show similar trends as the braking values were constant at the beginning 50 m and experience a similar rate of change when they ride approaching the curve entry point. This indicated that the closer the riders approached the curve, the higher the brake force applied as riders tended to reach a comfortable speed when they enter the curve section. Two regression equations were formed to study the change in brake intensity with the distances from curve entry for both braking systems and were shown as follows:

(2) $$\begin{array}{c} \text{Rear}\;\text{Brake}\;\text{Change}=0.1438\;\text{Distance}+12.102\\ \text{Front}\;\text{Brake}\;\text{Change}=0.1825\;\text{Distance}+14.032. \end{array}$$

It was found that the gradient for front brake regression line (0.1825) is steeper compared to the gradient on rear brake regression line (0.1438). This meant that front brake intensity rose at higher rate compared to the rear brake applied, where riders tend to increase the brake intensity to reduce the speed when entering into the curve section.

### 4.2. Structural Equation Modeling

A model was developed on the basis of structural equation modeling (SEM). Structural equation modeling is a multivariate technique that can be described as a combination of both factor analysis and path analysis. It is a statistical technique that allows the analyst to examine a series of dependence relationships between exogenous variables and endogenous variables simultaneously. An exogenous variable is one whose variability is assumed to be determined by causes outside the causal model under consideration. An endogenous variable, on the other hand, is one whose variation is to be explained by exogenous and other endogenous variables in the causal model.

**Figure 6:**
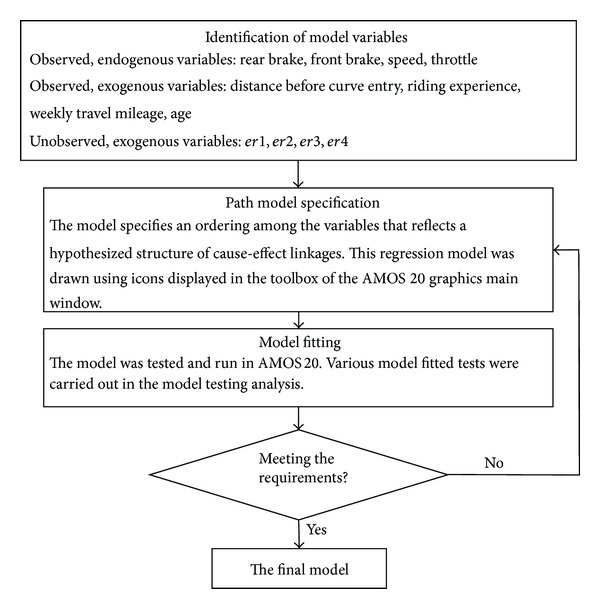
Framework showing the process of the development of SEM for riding behaviour before curve entry.

**Figure 7:**
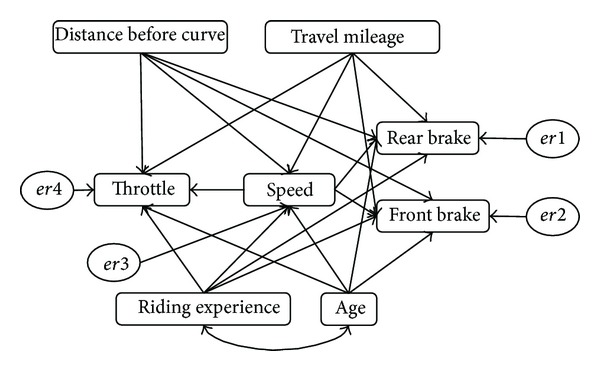
Path model for the prediction of riding behaviour before curve entry.

Model testing via path analysis can be carried out with the conventional multiple regression technique. That is, path coefficients can be estimated by regressing the endogenous variable (the dependent variable) onto the exogenous variables (the predictor variables) and then repeating the procedure by treating the exogenous variables as endogenous variables. Such a method, however, is typically piecemeal in nature and does not provide information regarding the hypothesized model's goodness-of-fit. Without information about the model's goodness-of-fit, it is difficult to assess the adequacy of the theory underlying the hypothesized model. SEM, on the other hand, is able to estimate the multiple and interrelated dependence relationships simultaneously. Because it tests the model as a whole, rather than in a piecemeal fashion, statistics can be calculated to show the goodness-of-fit of the data to the hypothesized model. Besides, conventional multiple regression technique assumes that the variables in the analysis are error-free while SEM improves statistical estimation by accounting for measurement error in the estimation process. In short, SEM analysis removed the potential biasing effects of the measurement error on the results and thus improved the statistical estimation process. The process of development of SEM path model for the study is shown in Figure 6.  Figure 7 illustrates the final path model for the entire riding behaviour study on curve entry.

The input covariance matrix generated from the model's eight observed variables contains 44 sample moments. For the hypothesized model (Figure 7), there are 36 parameters to be estimated. The model, therefore, has positive degrees of freedom (44 − 36 = 8), and the chi-squared goodness-of-fit was computed. The result indicates that the model did not fit the data well by chi-square test, *χ* ^2^(*N* = 1206, df = 8) = 399.157, *P* < 0.05. Although the hypothesized model did not fit the observed variance-covariance matrix well by the chi-square test, the baseline comparisons fit indices of NFI, IFI, and CFI are all above 0.9 (range: 0.921-0.922). These indices compare the fit of the hypothesized model to the null or independence model. Given the range of the computed baseline comparisons fit indices, the remaining possible improvement in fit for the hypothesized model (range: 0.078 to 0.079) appears so small as to be of little practical significance. In short, the model is sufficiently fit for the data. Since the original path model consists of four regression models, it is easier to study if we discuss and explain the model separately.

The covariance between “age” and “riding experience” is found highly significant by C.R. test (*P* < 0.001). The standardized correlation coefficient is found at 0.972, as shown in Table 3. Squared multiple correlation is an index of the proportion of the variance of the endogenous variable that is accounted for by the exogenous of predictor variables. It can be assumed that the higher the value of squared multiple correlation, the greater the explanatory power of the regression model and therefore the better the prediction of the dependent variable. Squared multiple correlations table shows that the percentage of variance explained range from 0.118 or 11.8% (speed) to 0.423 or 42.3% (throttle). The residual (unexplained) variances are calculated by subtracting each explained variance from 1. Thus, for the 4 measurement variables, the residual variances range from 57.7% to 88.2%.

Standardized coefficient estimates (*β*) are independent of the units in which all variables are measured. These standardized coefficients allow us to compare directly the relative relationship between each independent variable and the dependent variable. In the speed regression model, it was found that the standardized regression weights are all significant by the critical ratio test (>±1.96, *P* < 0.05) (see Table 3), except for “travel mileage,” where the C.R. = 1.063, *P* = 0.288. Based on this criterion, it can be seen that the variables “distance before curve,” “age,” and “riding experience” are highly significant predictors of speed (C.R. = 10.496, *P* < 0.001; C.R. = −4.095, *P* < 0.001; C.R. = 0.392, *P* < 0.001, resp.). From Table 3, the ratings on two variables of “distance before curve” and “riding experience” are both significantly and positively correlated to “speed” (*β* = 0.315; *β* = 0.392, resp.) while “age” is found negatively correlated to “speed.” Thus, it can be concluded that the speed reduced when they rode approaching the curve entry. Besides, it was found that the greater the riding experience is and the younger a rider is, the higher the riding speed is. In the throttle regression model, the standardized regression weights are all significant by the critical ratio test (>±1.96, *P* < 0.05). Based on this criterion, the variables “distance before curve,” “speed,” “age,” “riding experience,” and “travel mileage” are highly significant predictors of throttle. The ratings on two variables of “distance before curve” and “age” are both significantly and positively correlated to “throttle” (*β* = 0.641; *β* = 0.209, resp.) while “speed,” “riding experience,” and “travel mileage” are found negatively related to “throttle” (*β* = −0.398; *β* = −0.290; *β* = −0.063, resp.). In conclusion, the smaller the distance from the curve entry and rider's age, the lower the throttle force applied. In contrast, the greater the speed, riding experience, and travel mileage are, the smaller the throttle force applied by the riders will be.

In the rear brake regression model, the standardized regression weights are all significant by the critical ratio test (>±1.96, *P* < 0.05) that range from −0.567 to 0.269. These values indicate that the rear brake is significantly represented by “distance before curve,” “speed,” “age,” “riding experience,” and “travel mileage.” From Table 3, it can be seen that ratings on two variables of “distance before curve” and “riding experience” are both significantly and negatively correlated to the rear brake applied (*β* = −0.567; *β* = −0.502, resp.) while other variables are found positively correlated to rear brake. From this model, it was found that more rear brake force was applied when riding approaching curve and during higher riding speed. Meanwhile in the front brake regression model, the standardized regression weights are all significant by the critical ratio test (>±1.96, *P* < 0.05), except for variables “age” and “travel mileage” and range from −0.586 to 0.093. These values indicate that the front brake is significantly represented by “distance before curve,” “speed,” and “riding experience.” As observed from Table 3 also, it can be seen that ratings on two variables of “distance before curve” and “riding experience” are both significantly and negatively correlated to the front brake applied (*β* = −0.586; *β* = −0.217, resp.) while “speed” is positively correlated to front brake. In conclusion, more front brake force was applied in higher riding speed and at a distance closer to the curve entry point. The standardized regression equations for these models were shown as follows:

(3) Speed=0.315  Distance−0.477  Age+0.392  Riding  ExperienceThrottle=0.641  Distance −0.290  Riding  Experience +0.209  Age−0.398  Speed −0.063  Travel  MileageRear  Brake=−0.567  Distance−0.502  Riding  Experience+0.269  Age+0.071  Speed+0.117  Travel  MileageFront  Brake=−0.586  Distance−0.217  Riding  Experience+0.093  Speed.

## 5. Conclusions

In the riding study presented in this paper, it was found that the speed reduced in a different rate as riders ride approaching the curve section. The speed reduction rate can be expressed in a linear equation of *y* = −0.031*x* − 2.517, where *y* is the change in speed while *x* is the distance from curve entry. Besides, it was found that generally riders tend to apply higher rear brake compared to front brake at the whole riding procedure. Structural equation modeling (SEM) analysis is used to develop a model which suits the collected data in our case study. It is justified that “age” and “riding experience” are two correlated factors with the coefficient of correlation as 0.972. Multiple regression equation is formed for each of the regression models. Generally, it was found that distance from curve entry was the most significant and dominant factor that causes the change of throttle and brake force applied, which recorded high impact values, ranging from absolute value of 0.567 to 0.641. Other exogenous variables are found to have various rates of impact towards endogenous variables in each model set.

With the help of the instrumented motorcycle, the findings from this study should provide us with a brief idea on how riders would react and respond to the riding towards the curve section. The instrumented motorcycle is a very powerful tool as it provided a robust, accurate, and reliable way of collecting such important motorcycle riding data in this study of motorcyclist riding behaviour. By conducting such motorcycle accident and riders' behaviour studies, traffic engineers, law enforcers, and policy makers may take effective actions to reduce the number of accidents and therefore save valuable human life.

## Figures and Tables

**Table 1:** Fatality percentage by mode of transport in Malaysia.

Mode	2006		2007		2008		2009		2010	
	Fatal	%	Fatal	%	Fatal	%	Fatal	%	Fatal	%
Pedestrian	595	9.46	636	10.12	598	9.16	593	8.79	626	9.11
Bus	39	0.62	75	1.19	48	0.74	31	0.46	77	1.12
Lorry	257	4.09	232	3.69	207	3.17	225	3.34	213	3.10
Motorcar	1248	19.85	1271	20.23	1371	21.01	1446	21.44	1477	21.49
Motorcycle	3693	58.74	3646	58.04	3898	59.72	4070	60.34	4036	58.73
Others	455	7.24	422	6.72	405	6.20	380	5.63	443	6.45

Total	6287	100.00	6282	100.00	6527	100.00	6745	100.00	6872	100.00

**Table 2:** Number of vehicle crashes by mode of transport in Malaysia.

Mode	2006		2007		2008		2009		2010	
	No.	%	No.	%	No.	%	No.	%	No.	%
Bus	1,080	2.33	700	1.55	615	1.48	531	1.29	410	1.10
Lorry	3,728	8.04	3,595	7.94	3,296	7.93	3,199	7.79	2993	8.00
Motorcar	17,068	36.81	16,110	35.56	14,827	35.68	15,196	37.01	13,630	36.44
Motorcycle	20,717	44.68	21,408	47.25	19,758	47.54	19,416	47.29	17,827	47.66
Others	3,774	8.14	3,491	7.71	3,064	7.37	2,713	6.61	2,548	6.81

Total	46,367	100.00	45,304	100.00	41,560	100.00	41,055	100.00	37,408	100.00

**Table:** (a) Regression weights and standardized regression weights

	Unstandardized coefficient est.	Standardized coefficient est.	S.E.	C.R.	*P*
Speed*←*distance	0.091	0.315	0.009	10.496	∗∗∗
Speed *←* age	−0.472	−0.477	0.115	−4.095	∗∗∗
Speed *←* riding exp.	0.380	0.392	0.113	3.372	∗∗∗
Speed *←* travel mileage	0.002	0.029	0.001	1.063	0.288
Throttle *←* distance	0.293	0.641	0.012	24.690	∗∗∗
Rear brake *←* speed	0.283	0.071	0.103	2.759	0.006
Front brake *←* speed	0.347	0.093	0.100	3.468	∗∗∗
Throttle *←* riding exp.	−0.445	−0.290	0.152	−2.919	0.004
Throttle *←* travel mileage	−0.005	−0.063	0.002	−2.721	0.007
Rear brake *←* riding exp.	−1.947	−0.502	0.394	−4.940	∗∗∗
Front brake *←* riding exp.	−0.788	−0.217	0.384	−2.050	0.040
Rear brake *←* travel mileage	0.025	0.117	0.005	4.921	∗∗∗
Front brake *←* travel mileage	0.000	−0.001	0.005	−0.042	0.966
Throttle *←* age	0.327	0.209	0.156	2.097	0.036
Rear brake *←* age	1.067	0.269	0.404	2.645	0.008
Front brake *←* age	0.509	0.137	0.394	1.293	0.196
Rear brake *←* distance	−0.658	−0.567	0.031	−21.225	∗∗∗
Front brake *←* distance	−0.636	−0.586	0.030	−21.041	∗∗∗
Throttle *←* speed	−0.628	−0.398	0.040	−15.792	∗∗∗

Note: ***indicate that the probability value is less than 0.001.

**Table:** (b) Variance

	Covariance	Correlation coefficient	S.E.	C.R.	*P*
Riding exp.*↔* age	110.098	0.972	4.550	24.195	∗∗∗

**Table:** (c) Squared multiple correlations

	Estimate
Speed	0.118
Throttle	0.423
Front brake	0.326
Rear brake	0.379
